# Modelling neuroanatomical variation during childhood and adolescence with neighbourhood-preserving embedding

**DOI:** 10.1038/s41598-017-18253-6

**Published:** 2017-12-19

**Authors:** Gareth Ball, Chris Adamson, Richard Beare, Marc L. Seal

**Affiliations:** 1 Developmental Imaging, Murdoch Children’s Research Institute, The Royal Children’s Hospital, Melbourne, Australia; 2 Department of Paediatrics, University of Melbourne, Melbourne, Australia

## Abstract

Brain development is a dynamic process with tissue-specific alterations that reflect complex and ongoing biological processes taking place during childhood and adolescence. Accurate identification and modelling of these anatomical processes *in vivo* with MRI may provide clinically useful imaging markers of individual variability in development. In this study, we use manifold learning to build a model of age- and sex-related anatomical variation using multiple magnetic resonance imaging metrics. Using publicly available data from a large paediatric cohort (n = 768), we apply a multi-metric machine learning approach combining measures of tissue volume, cortical area and cortical thickness into a low-dimensional data representation. We find that neuroanatomical variation due to age and sex can be captured by two orthogonal patterns of brain development and we use this model to simultaneously predict age with a mean error of 1.5–1.6 years and sex with an accuracy of 81%. We validate this model in an independent developmental cohort. We present a framework for modelling anatomical development during childhood using manifold embedding. This model accurately predicts age and sex based on image-derived markers of cerebral morphology and generalises well to independent populations.

## Introduction

Brain development is a dynamic process that follows a well-defined trajectory during childhood and adolescence. Variations in neurodevelopmental trajectories over this critical period have direct consequence for adult functioning and mental health. During this formative period the brain undergoes profound change: increases in brain size, most rapid after birth, continue into late adolescence^1^; myelination processes that begin *in utero* continue to progress through to the second decade of life^2^, and synaptic pruning leads to significant reductions in synaptic density during early adolescence^3^. Magnetic resonance imaging (MRI) provides the opportunity to study brain development and track these developmental processes *in vivo*.

Analyses of structural MRI have found that grey and white matter volumes follow different trajectories during adolescence. Cortical grey matter volume peaks in childhood, then gradually decreases during adolescence^4,5^, whereas white matter volume increases throughout childhood and adolescence^6,7^. These observations were recently confirmed across four independent samples, where Tamnes *et al*. observed consistent developmental trajectories characterised by a decreasing cortical thickness with increasing age and childhood increases in surface area followed by subtle decreases during adolescence^8^. Evidence suggests that sexual dimorphism may also play a role in cerebral development. Sex differences have been observed in developmental studies of cortical thickness^9^ and sex-by-age interactions in area and folding have been reported in frontal and temporal cortex in adolescence^10,11^. These differences may result from delays in development along similar trajectories, the timing of which can coincide with pubertal onset^12,13^, although the accurate definition and timing of developmental peaks is difficult and may be at risk to potential model or sample biases^14,15^. Any differences between the sexes are likely subtle; male brains are larger throughout development^16^, and regional, volumetric estimates of sex differences can be diminished once corrected for global differences in scale^4^.

Taken together, these studies present a consensus view of typical cerebral development. However, longitudinal studies of healthy populations have shown that individual development can deviate significantly from these canonical trajectories^15^, highlighting the need to create models of typical growth and development of the brain that can be applied on an individual level. Establishing developmental trajectories for typical cerebral development is vital to our understanding of maturational brain change and may provide a more accurate understanding of relationships between brain maturation and behavioural phenotypes during development.

MRI data can generally be considered high-dimensional: a single image, or cortical surface, may comprise over 250,000 voxels, or vertices, many of which are highly correlated and interrelated. Manifold learning refers to a suite of dimension reduction techniques based on the intuition that high-dimensional datasets (such as MRI) reside on an embedded low-dimensional manifold or subspace. The aim is to learn a mapping between the high- and low-dimensional data representations while preserving certain statistical properties (e.g.: variance, community structure) of the original data. Such methods are well established in computer vision problems^17–20^ and bioinformatics^21–23^ to deal with high-throughput data and can allow for an easier and more intuitive interpretation of important model features while retaining the underlying nonlinear relationships present between individual datapoints in the original dataset.

**Figure 1:**
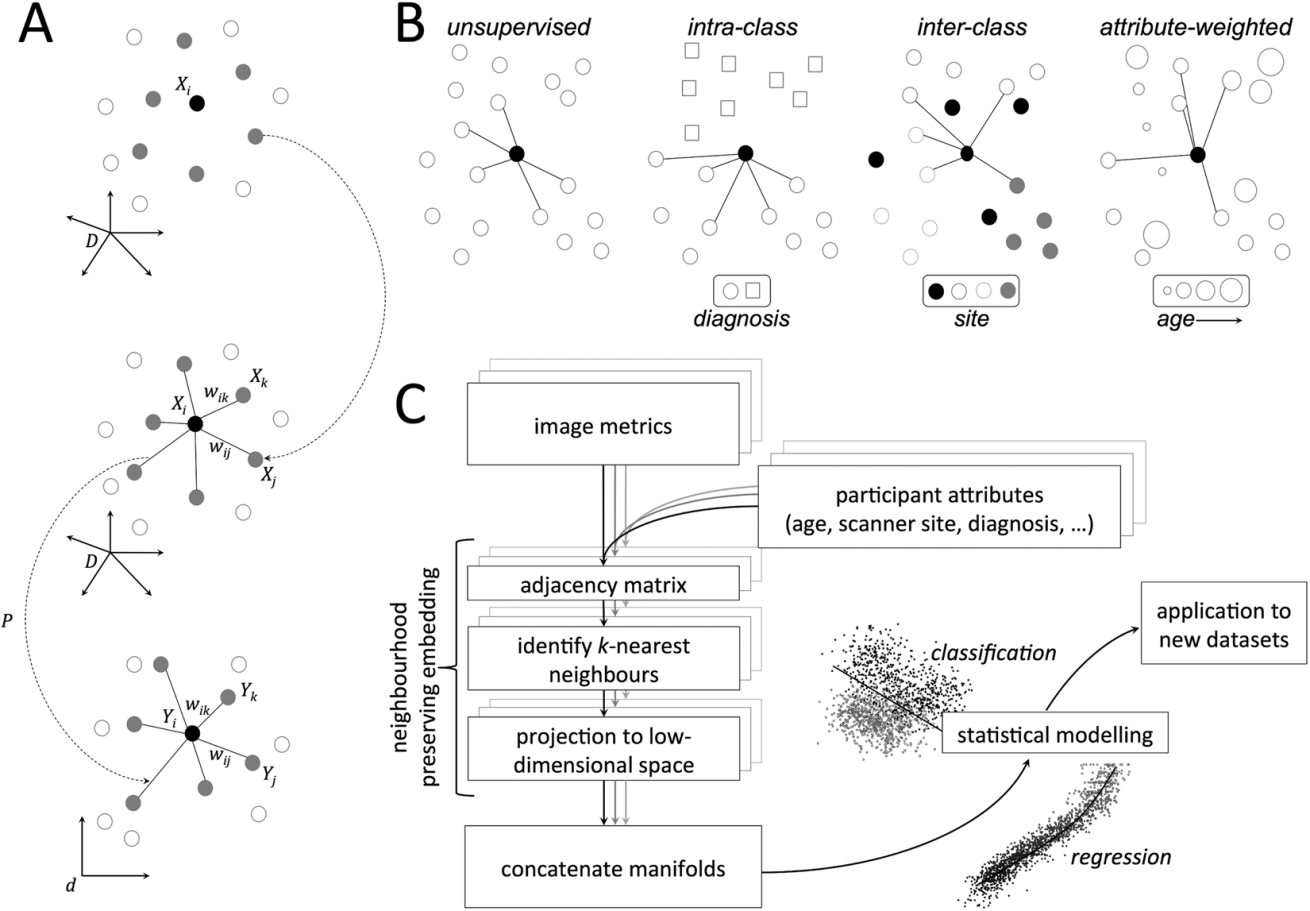
Neighbourhood preserving embedding. (**A**) For a given datapoint, ${X}_{i}$, nearest neighbours are selected and weights assigned that can be used to approximately reconstruct ${X}_{i}$. A linear transformation, *P*, is then sought to project the data into a low-dimensional space while preserving the neighbourhood structure. (**B**) Possible supervision strategies for neighbourhood construction. In an unsupervised setting, neighbours are selected based on image similarity alone; alternatively, neighbours can be selected from within- or between-classes in order to maximise/minimise group differences in the manifold structure. Similarly, neighbours can be selected based on the weighted combination of image similarity and that of another subject-specific attribute (e.g.: age). (**C**) Analysis pipeline for NPE analysis. For each image metric, NPE is used for subspace projection, before the embedded data are combined and passed on for statistical modelling.

One such example, Neighbourhood Preserving Embedding (NPE) aims to preserve the local neighbourhood structure of the data once projected onto the manifold^17^. That is, communities, patterns, or between-group differences that exist among datasets in the high-dimensional setting are maximally preserved within the low-dimensional subspace. Local neighbourhoods are typically defined based on the Euclidean distance between samples in the high-dimensional image space, however it is possible to introduce other constraints in order to conduct NPE in a supervised setting (Fig. 1)^17,24^. For example, restricting local neighbourhoods to only include subjects from the same class, or diagnostic group, to enhance separation in the low-dimensional space.

Manifold learning methods have great potential to aid in the analysis and understanding of high-dimensional MRI datasets^25–27^ with recent examples demonstrating improved discrimination between neurological disease states^28^, mapping of developmental trajectories in the newborn brain^29^, and detection of white matter lesions^30^. Dimension reduction leverages the redundancy often present in high-dimensional data, acting as a form of statistical regularisation and reducing the risk of overfitting models when the number of samples is much less than the number of model features. Importantly, these methods can serve to highlight latent patterns within the dataset that align with variables of interest such as age, sex or clinical diagnosis.

Recently, studies have shown that it is possible to accurately predict an individual’s age^31–35^ or sex^36,37^ based on MRI images alone. These methods involve the use of modern multivariate techniques to extract informative morphological features to act as neuroanatomical markers of age or sex in a predictive model. In this study, we aim to build on these observations, introducing supervised NPE as a method to isolate structured patterns of covariance within populations, and simultaneously modelling typical neuroanatomical variation due to age and sex during development. Using a multi-metric approach, we combine measures of tissue volume, cortical area and cortical thickness to build a model that predicts age and sex with high accuracy and generalises well to other developmental populations.

## Results

For every participant (n = 768, age 3.0–21.0 y), we constructed maps of brain tissue volume, cortical thickness and cortical area from T1-weighted MRI (see Methods). For each image metric (volume, thickness and area), we projected the full image data matrix to a 3 dimensional manifold using NPE based on 10 nearest-neighbours (Fig. 1). In order to maximally preserve age- and sex-related variation in the embedded data, we incorporated participant attributes into the construction of the weighted neighbourhood matrices (Fig. 1B; see Methods).

**Figure 2:**
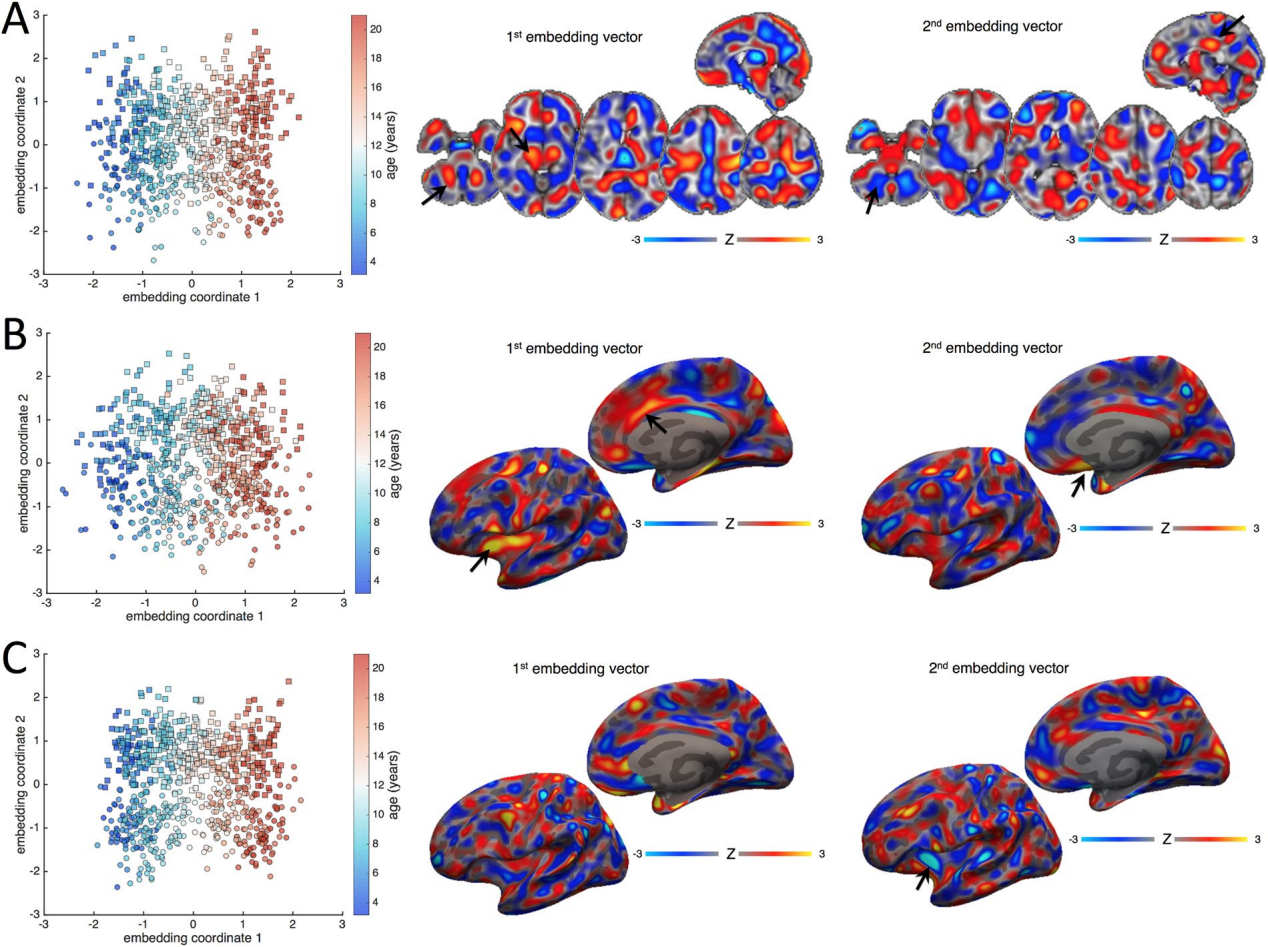
Manifold structure for tissue volume, cortical area and cortical thickness. Manifold structure is visualised for tissue volume (**A**), cortical area (**B**) and cortical thickness (**C**). For each image metric, the first two embedding coordinates are plotted against each other. Each point represents a subject; the colourbar indicates age and markers denote sex (square: male; circle: female). Images show the embedding vectors for the first and second coordinates, i.e.: the model coefficients in each voxel required to transform data into the embedded subspace. Maps are Z-scored for comparison (colourbar).

Figure 2 shows manifold structure visualised for tissue volume (Fig. 2A), cortical area (Fig. 2B) and cortical thickness (Fig. 2C) calculated in the PING cohort. For each metric, an orthogonal rotation was applied to each manifold to maximise correlation with age and sex along the first and second axes respectively. The images show the (standardised) weight of the embedding vectors (model coefficients) used to project new data to the rotated manifold, thus important features are represented by a larger weight, and increases in volume, thickness or area in regions with high positive weight will result in a positive increase along the respective embedding axis and vice versa.

For all three modalities, age increases along the first embedding coordinate, indicated by the gradation of colour along the first axis of the scatter plots Increasing age (a positive embedding coordinate) is associated with a neuroanatomical pattern represented by relatively increased tissue volume in the cerebellum, brain stem and thalamus (black arrows; Fig. 2A), and ascending white matter tracts subjacent to the primary motor cortex (positive image weights), with relative decreases in medial frontal and parietal cortices (negative image weights, Fig. 2A). In the cortex, age is associated with increasing surface area in the insula and anterior cingulate cortex (black arrows, Fig. 2B).

Separation between sexes is shown along the second dimension (squares and circles, Fig. 2). This is associated with a distributed, discriminant pattern of tissue volume alterations with increases in medial posterior cingulate (black arrow; Fig. 2A) and primary visual cortex, and brainstem, and relative decreases in the basal ganglia, frontal pole, and cerebellum (negative weights, black arrow) associated with male sex. Separation along the second dimension was also associated with regions of increased surface area in medial temporal and orbitofrontal cortices (black arrow, Fig. 2B), and decreased cortical thickness in the anterior insula (arrow, Fig. 2C).

**Figure 3:**
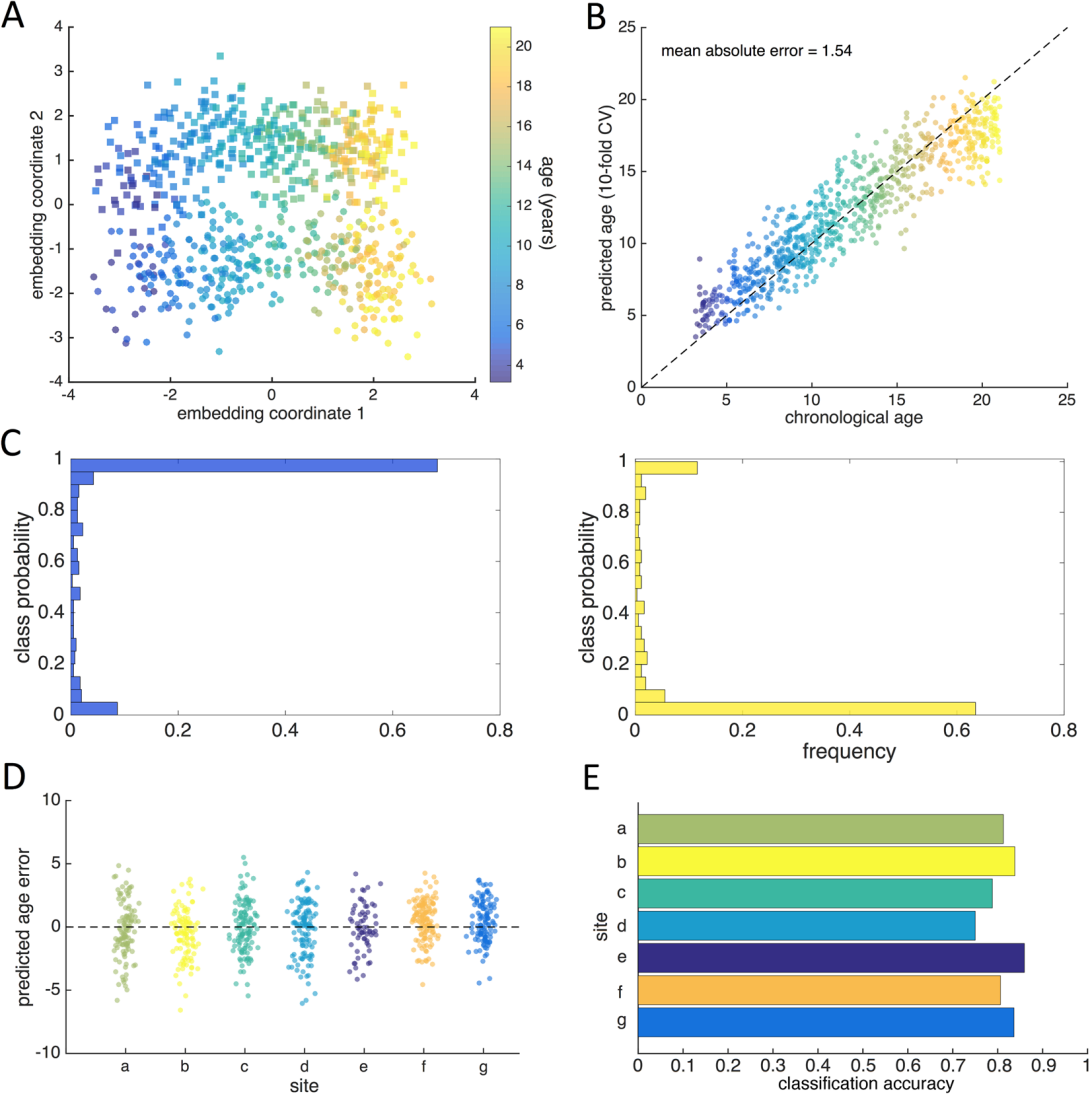
Age and sex prediction with manifold embedding. (**A**) The first two coordinates of the joint manifold are shown, each point represents a subject; the colourbar indicates age and markers denote sex (square: male; circle: female). (**B**) Using 10-fold cross-validation, age and sex were predicted based on the concatenated manifold coordinates. Gaussian Process regression was used to predict age, shown plotted against chronological age (colourbar shown as in **A**). (**C**) Predicted class probabilities are shown for males (blue histogram) and females (yellow). (**D**) Predicted age error is shown for each site in PING separately. (**E**) Sex classification accuracies for each site in PING.

Figure 3A shows the joint manifold structure visualised after concatenating all three image metrics and performing a final dimension reduction on the concatenated coordinate data, ${Y}_{c}=({Y}_{v},{Y}_{t},{Y}_{a})$ using PCA^29^. This demonstrates how individual variation associated with age and sex during development can be captured along two orthogonal dimensions in this population.

### Model accuracy

Using 10-fold cross-validation, we predicted age in the PING cohort with a mean absolute error (MAE) of 1.54 years (correlation between chronological and predicted age = 0.926; Fig. 3B). Using a linear discriminant classifier, we predicted sex with an accuracy of 80.9% (Fig. 3C).

Our model was robust to site variation in the PING cohort: performing NPE without the additional site constraint did not affect the prediction accuracies (MAE = 1.49 y, accuracy = 80.0%) and there was no significant association between acquisition site and absolute age estimation error (ANOVA: F_6,761_ = 1.95, p = 0.07; Fig. 3D). Classification accuracy was similar across sites, ranging from 75.0–85.9% (Fig. 3E).

**Figure 4:**
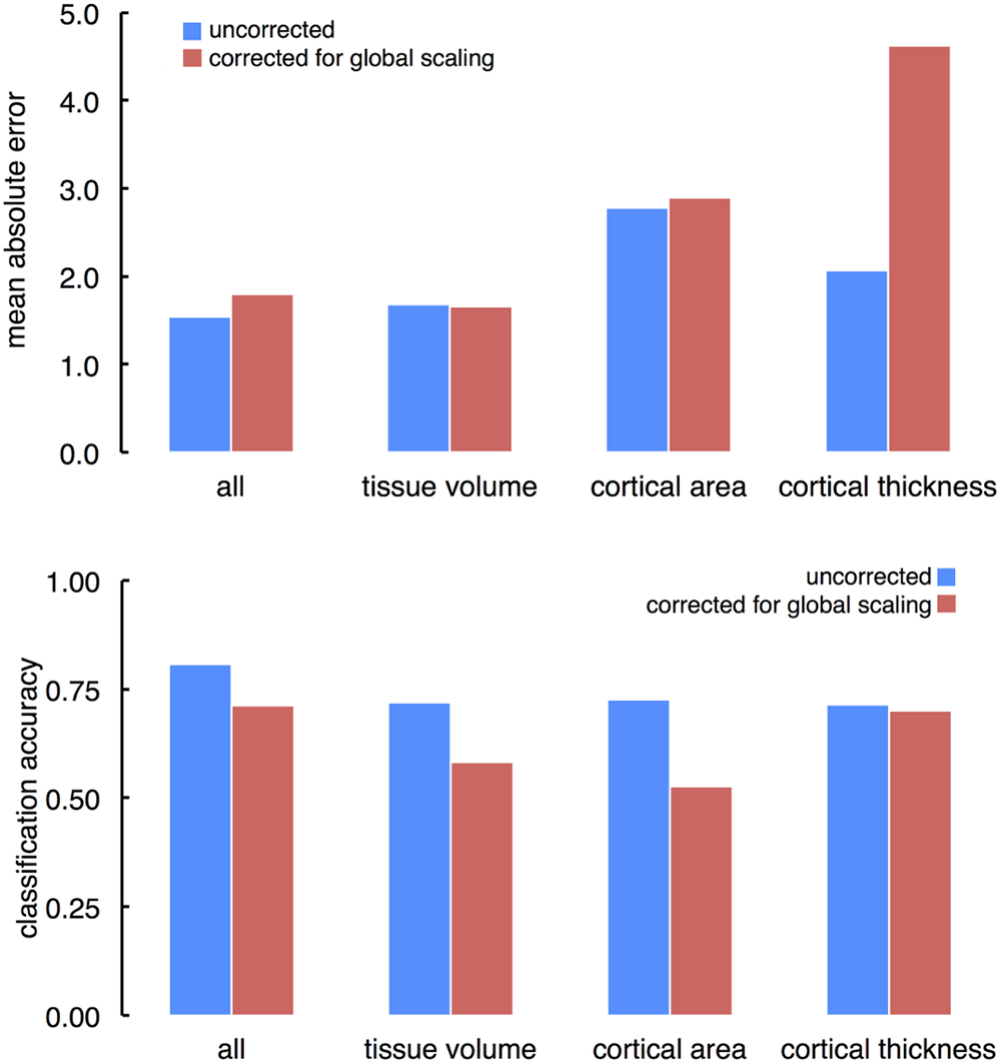
Age and sex prediction after correction for global scaling. (Top) Mean absolute error for the full manifold and for each imaging metric are shown before and after correction for individual differences in global scale. (Bottom) Corresponding sex classification accuracies for the full manifold and for each imaging metric.

We repeated this analysis, accounting for differences in global scale by correcting voxel-(vertex-)wise measures of tissue volume, cortical surface area and thickness metrics for intracranial volume, total surface area and mean cortical thickness, respectively. After correcting for global scale, we achieved a cross-validated MAE of 1.79 years, and an accuracy of 71.4% in the PING cohort (Fig. 4).

### Contribution of each metric

To assess the individual contribution of each set of image metrics to the final model, we also performed the analysis using only tissue volume, cortical thickness or cortical area data. We found that the joint model combining all three metrics outperformed single metric models for both age and sex prediction (tissue volume only: MAE = 1.69, accuracy = 71.9%; area: MAE = 2.77, accuracy = 72.7%; thickness: MAE = 2.07, accuracy = 71.5%). Figure 4 shows MAE and classification accuracies for each tissue metric with and without correction for variations in global scale.

After correcting for variation due to global scale, the ability to discriminate between sexes was reduced for tissue volume (classification accuracy = 58.3%) and cortical area (52.7%), but not cortical thickness (70.1%). In contrast, global scaling significantly increased error in age prediction estimates based on cortical thickness (MAE = 4.62 y), but had little impact on models using tissue volume (1.66 y) or cortical area (2.96 y).

### Associations between model accuracy and age

To determine if model accuracy varied with age, we partitioned our data into 10 approximately equal-sized bins and calculated MAE and classification accuracy in each (Table 1). Age prediction error ranged from a minimum of 1.23 y at around 9 years of age, to a maximum of 2.55 y in the oldest participants (mean age = 20.3 y). In contrast, classification accuracy ranged from 69% to 87%, with discrimination lowest in the youngest participants (mean age = 4.5 y) and highest at around 16 years. After correction for global scale, the minimum MAE was 1.50 (mean age 11.7 y; maximum MAE = 2.9, mean age 22.3 y), and the lowest and highest classification accuracies were 65.3% in the youngest group, and 80.3% at 16 years.

**Table 1:** Age and sex prediction accuracy at different ages.

**Bin**	**n**	**mean age**	**MAE**	**male (%)**	**accuracy**
1	75	4.48	1.69	38 (50.1)	69.33
2	79	7.31	1.32	44 (55.7)	79.75
3	71	8.96	1.33	38 (53.5)	78.87
4	79	9.63	1.23	35 (44.3)	82.28
5	77	11.72	1.40	43 (55.8)	81.82
6	80	12.89	1.46	52 (65.0)	82.50
7	77	14.52	1.29	47 (61.0)	81.82
8	76	16.15	1.46	36 (47.4)	86.84
9	74	17.63	1.62	34 (46.0)	81.08
10	80	20.29	2.55	38 (47.5)	83.75

### Associations between model accuracy and cognition

In order to determine if deviations from the average developmental trajectory of the brain coincided with differences in cognitive performance we compared predicted age errors (the difference between age estimated from MRI using the above model and true, chronological age) with cognitive scores in PING.

In the PING cohort, no significant associations were found between NTCB scores (corrected for age, sex, socioeconomic status and genetic ancestry) and predicted age error after correcting for multiple comparisons (Table 2).

**Table 2:** Associations between predicted age error and cognitive score in PING.

**NTCB score**	**Linear regression**		**p**
	**R** ^**2**^	**F**_**1,615**_	
Flanker	0.000	0.018	0.892
Attention	0.000	0.068	0.794
Picture Sequence Memory	0.007	4.292	0.039
List Sorting	0.001	0.469	0.494
Picture Vocabulary	0.003	2.118	0.146
Reading	0.000	0.072	0.789
Dimensional Change Card Sorting	0.001	0.315	0.575
Pattern Comparison	0.007	4.247	0.040

### Alternative parameter settings

Prediction accuracies in the PING cohort were robust to altering the number of neighbours, *k*, used in manifold construction (*k* = 5: classification accuracy = 80.9%, MAE = 1.51 y; $k$ = 20: accuracy = 79.7%; MAE = 1.53 y), and the number of manifold dimensions, *d* (*d* = 5: accuracy = 80.1%, MAE = 1.59 y; $d$ = 10: accuracy = 81.1%, MAE = 1.59 y).

Finally, we found that using NPE for subspace projection outperformed dimension reduction with PCA (MAE = 1.61 y, accuracy = 71.2%; correcting for global scaling, MAE = 1.91 y, accuracy = 54.4%).

### External model validation

To test the external validity of this modelling approach, we projected imaging data acquired from typically developing controls in two independent populations (ABIDE and ABIDE-II) onto a low-dimensional manifold constructed from the PING dataset. Using this approach, we predicted age in ABIDE with an MAE of 1.65 years (correlation: 0.825; Fig. S1A), and sex with an accuracy of 80.0% (Fig. S1B; 1.87y and 71.2% after correction for global scaling). We achieved similar results in ABIDE-II (Fig. S1; MAE = 1.54, correlation: 0.817; accuracy = 80.2; 1.80 y and 70.6% after correction for global scaling). In contrast to the PING data, a main effect of site was evident in both ABIDE and ABIDE-II age prediction error (F_16,407_ = 1.86, p = 0.02; F_14,424_ = 16.56, p < 0.001; Supplemental Information; Fig. S1C,D). When considering MAE and accuracy over sites in ABIDE and ABIDE-II, we find a positive correlation between mean cohort age and MAE (i.e: age prediction is worse in sites with older participants; ABIDE r = 0.23, *ns*; ABIDE II r = 0.78, p < 0.001), however, as in the PING cohort, we see an improvement in sex prediction accuracy with increasing age (ABIDE r = 0.72, p < 0.001; ABIDE-II r = 0.22 *ns*).

## Discussion

In this study, we use manifold learning to generate a parsimonious description of typical brain development during childhood and adolescence. By combining measures of tissue volume, cortical thickness and cortical area, we show how patterns of anatomical variation can be used to accurately predict age and sex between the ages of 3 and 21 years. We show that this model is not strongly affected by site-to-site variation in image acquisition and yields accurate predictions across different study populations.

We demonstrate that supervised NPE can be used to predict biological age from MRI with a mean error of around 1.5 years. This is in line with previous reports in this population. Using a set of 231 pre-selected, image-based features from T1, T2 and diffusion-weighted MRI, Brown *et al*. developed a nonlinear model of cerebral maturation to predict age in the PING cohort, achieving an MAE of 1.03 years^31^. Using just T1-weighted image features, Brown *et al*. reported an MAE of 1.71 y, comparable to the MAE reported in this study. Using a similar approach, Franke *et al*. reported an MAE of 1.1 years in a cohort aged 5 to 18^38^. Using nonlinear mapping functions (i.e.: kernels) in machine learning allows for the use of linear methods to discover highly nonlinear boundaries or patterns in the original data by creating an implicit feature space^39^. While flexible, a limitation of these methods is the inability to identify important features in the space of the original dataset. An alternative approach may be to use regularised linear regression across all cortical regions, although this method depends upon an initial cortical parcellation^35^. By calculating a linear mapping between the original, high-dimensional data and the low-dimensional embedded manifold, NPE produces a set of basis vectors that – through linear combination – can approximately reconstruct the original dataset, capture nonlinear relationships and provide interpretable voxel-(vertex-)wise maps of feature importance^17^.

We show examples of these vectors in Fig. 2 for each image metric. The images represent distributed patterns of neuroanatomical variation that highlight regional importance within the model. As reported previously, increasing age is reflected a lower-to-higher-order trajectory characterised by reduced tissue volume in frontal and parietal regions, coupled with increased white matter tissue and brain stem volume^6,40,41^. Increasing age was also associated with increased cortical surface area most prominent in the insula and cingulate cortex. This agrees with previous reports of high rates of cortical surface area expansion during childhood in regions associated with higher-order intellectual function^42,43^.

We also find that male sex is predicted by a pattern of neuroanatomical variation including increased brain tissue volume in the posterior cingulate and occipital lobe, volumetric decreases in the basal ganglia and insula, alongside a pattern of reduced cortical surface area in medial frontal regions and decreased cortical thickness in the insula. We highlight that the manifold embedding coordinates defined by NPE are orthogonal by construction; as such, the patterns shown in Fig. 2 reflect anatomical variation independently associated with age and sex during development.

Sexual dimorphism during development is a contentious issue. Developmental trajectories for cortical grey and white matter appear similar between sexes^4,6^ with perceived sex differences often assigned to variation in physical size^1,44^. In a longitudinal study of 387 subjects aged 3 to 27, Lenroot *et al*. reported increased frontal grey matter volume in females and increased occipital white matter in males, after accounting for brain size^13^. Conversely, Sowell *et al*. reported thicker parietal and posterior temporal cortex in females, independent of age^9^. These discrepant findings may reflect the different timing of puberty or the differential effects of testosterone on brain development males and females^45^. In addition, male brains are larger than females throughout development^16^, and correcting for differences in global scale can diminish perceived sex differences in regional, cerebral volumes^4^. After correcting for global differences in intracranial volume, total surface area and mean cortical thickness, we were still able to achieve relatively accurate predictions of age and sex across development, although both were maximised with the inclusion of global scaling information.

Concerning differences in global scale that vary with age or sex, we report accuracies of both corrected and uncorrected metrics, using ICV, total surface area and mean cortical thickness as covariates. Previous developmental studies have used ICV, or alternatively brain tissue volume (specifically: parenchymal volume, excluding ventricular CSF) to correct regional measures of volume, both of which have advantages and disadvantages^15^. As a consensus on the use of ICV or brain tissue volume to correct volumetric measures is lacking^4^, we report uncorrected and ICV-corrected accuracies, though we note that brain tissue volume and ICV correlated strongly in the PING cohort (r = 0.87) and our model performance was not affected by the use of brain tissue as a covariate instead of ICV.

Importantly, our study confirms recent reports that multivariate analyses that consider whole-brain patterns of variation in brain morphometry can reliably and accurately discriminate sex, even if large within-class, or between region, variability exists^36,37^. We also show that this dimorphic pattern is evident even at very young ages, achieving a classification accuracy of around 69% (65% after global correction) in the youngest participants (aged 3–5 y). It is important to consider that this finding does not imply that all females have e.g.: a smaller posterior cingulate than all males, or even that the cingulate is on average smaller in females across populations. We suggest that this pattern of variation is one of a number (including the pattern of age-related variation described above) that exist concurrently within a population. An individual’s anatomical phenotype can then be viewed as arising from the weighted expression of these patterns, with the weight dependent on e.g.: age, sex, environmental or genetic factors. As such, tissue volume or cortical thickness, measured at a single point reflects the combination of multiple, distributed patterns of variation and, as such, may vary greatly over a population and not necessarily in line with a given covariate of interest (e.g.: sex).

In contrast to previous reports, we do not find strong evidence that deviation from a predicted developmental trajectory corresponds to adverse functional or behavioural outcome in healthy individuals. Previously, the difference between chronological age and age predicted from neuroimaging has been framed as an index of accelerated or delayed development or aging, depending on the direction of the discrepancy^46^. Recently, Erus *et al*. reported that a significant increase in predicted brain age compared to chronological age in childhood (predicted by a multi-modal MRI assessment) was indicative of precocious cognitive development (and *vice versa*), suggesting that complex cognitive phenotypes could be captured as variation along a single dimension of brain development^33^. In this study, we did not find any statistically significant associations between measures of cognitive function and age prediction error in typically-developing individuals. In the PING cohort, Akshoomoff *et al*., found that age, sex, socioeconomic status and genetic ancestry explained between 57% and 73% of variance in each of the NCTB scores^47^. After correcting for these factors, we found that brain age estimation error explained at most 0.7% of the remaining variance (in memory and pattern comparison tests).

This suggests that model error in age prediction in this context does not reflect the impact of an underlying latent variable associated with cognition. This discrepancy is likely due to differences in model construction between methods. Here, we use NPE to extract a single dimension of anatomical variation that aims to maximally preserve specific age-related structure in the data. As such, the reported pattern may lie orthogonal to neuroanatomical correlates of (non age-related) cognitive performance and as mentioned above, such patterns, while coexistent within the population, will not vary as a function of each other. Indeed, once corrected for age-related variation, measures of cognition will, by definition, lie orthogonal to age, and thus age-specific neuroanatomical variation. Other methods that predict age based on the appearance of the brain as a whole, might better reflect the conflation of cognitive and age-related ‘components’ during development, such that model error captures variation in anatomy aligned with cognition^33^.

We found that age prediction accuracy decreased slightly with increasing age. In the PING cohort, MAE was lowest at around 9 years, and highest at 20. Other studies have found similar discrepancies^35,48^ and this may indicate the increasing difficulty of discrimination between individuals in early adulthood compared to childhood and adolescence, suggesting that image-based prediction is most accurate during the periods when the rate of anatomical change is greatest^1,31^.

In an effort to assess the external validity of this approach, we tested the accuracy of the NPE model constructed in the PING dataset to predict age and sex in a comparative population. Overall, we found this model was able to age and sex to the same level of accuracy in the ABIDE cohorts (Fig. S1A,B). We also observed a trend towards a model-based underestimation of age in the older participants. However, although no effect of site was found in the PING cohort, there was a significant effect of site on accuracy in both ABIDE and ABIDE-II (Fig. S1). This effect appeared to be compounded by the uneven distribution of age across sites, with the mean age within site ranging from 8.15 years to 20.75 years (Table S2), and the older sites showing significantly poorer model performance (e.g.: site *p* in ABIDE; sites *a* and *k* in ABIDE-II; Fig. S1).

Our findings suggest that the anatomical maturation of the brain during childhood and adolescence can be accurately modelled within a low-dimensional subspace. That is, variation along two axes is sufficient to capture individual variations due to age and sex within a population with relatively high accuracy. In contrast, functional or cognitive development is not well represented by variation along these axes. This suggests that additional, orthogonal dimensions of development are required to more accurately model individual trajectories. Alternatively, this approach may benefit from incorporating information from additional imaging modalities (e.g.: functional MRI) in order to more fully capture phenotypic variation associated with cognitive development^33,49^.

Subspace projection methods, such as NPE, bring focus to reliable and robust patterns that can predict phenotypic characteristics based on the brain’s shape and appearance. Global, linear methods including PCA and ICA have proven highly effective in identifying patterns in MRI data analysis^27,50,51^. However, as PCA seeks to preserve global properties of the data it may be dominated by sources of confounding variance, e.g.: site-to-site variation^52^. Alternatively, nonlinear embedding methods can often achieve highly-accurate embeddings based on local geometry, allowing visualisation of complex geometry in low-dimensional space but can not be easily applied to new, unseen datapoints^19,53^. NPE somewhat combines these approaches, calculating a linear embedding that allows the projection of unseen data into a previously defined subspace, learned from local neighbourhood geometry. In a clinical setting, this framework could be extended to explore anatomical patterns underlying developmental or neuropsychiatric disorder, or stratifying clinical populations by locating individuals within clusters based on the expression of different neuroanatomical imaging components. Indeed, combining functional, diffusion and possibly genetic information into a larger manifold framework and considering similarities over multiple modalities to model local neighbourhoods and communities within large datasets could provide a more complete model of individual variation during this time period. In addition, the projection of longitudinal data onto the manifold could enable individuals to be tracked over time, an important consideration for developmental studies^15^. With increasing sample sizes from large-scale imaging studies, there is also great potential for the application of deep learning to such problems^54,55^.

In summary, we present a framework for modelling anatomical development during childhood. This model accurately predicts age and sex based on image-derived markers of cerebral morphology and generalises well to independent populations.

## Methods

### Imaging data

To model typical neurodevelopment, 3 Tesla, T1-weighted MRI data were obtained from the PING Study^56^. The PING cohort comprises a large, typically-developing paediatric population with participants from several US sites included across a wide age and socioeconomic range. The human research protections programs and institutional review boards at all institutions (Weil Cornell Medical College, University of California at Davis, University of Hawaii, Kennedy Krieger Institute, Massachusetts General Hospital, University of California at Los Angeles, University of California at San Diego, University of Massachusetts Medical School, and Yale University) participating in the PING study approved all experimental and consenting procedures, and all methods were performed in accordance with the relevant guidelines, regulations and PING data use agreement^31^. Written parental informed consent was obtained for all PING subjects below the age of 18 and directly from all participants aged 18 years or older. Exclusion criteria included: a) neurological disorders; b) history of head trauma; c) preterm birth (less than 36 weeks); d) diagnosis of an autism spectrum disorder, bipolar disorder, schizophrenia, or mental retardation; e) pregnancy; and f) daily illicit drug use by the mother for more than one trimester^56^. Similar proportions of males and females participated across the entire age range.

The PING cohort included 1493 participants aged 3 to 21 years, of whom 1249 also had neuroimaging data. Of these, n = 773 were available to download from NITRC (https://www.nitrc.org). T1 images were acquired using standardized high-resolution 3D RF-spoiled gradient echo sequence with prospective motion correction (PROMO) at each site, with pulse sequences optimized for equivalence in contrast properties across scanner manufacturers (GE, Siemens, and Phillips) and models^56^.

In addition to imaging data, participants undertook comprehensive behavioural and cognitive assessments (NIH Toolbox Cognition Battery, NTCB^47^; and provided a saliva sample for genome-wide genotyping. The NTCB comprises seven tests (Flanker, Picture Sequence, List Sorting, Picture Vocabulary, Reading, Dimensional Change Card Sorting, Pattern Comparison) that measure abilities across six major cognitive domains, including cognitive flexibility, inhibitory control, and working memory^47^.

### External validation data

For model validation, a comparative neurodevelopmental population was obtained from the ABIDE and ABIDEII datasets^57,58^. These datasets represent a consortium effort to aggregate MRI datasets from individuals with autism spectrum disorder and age-matched typically-developing controls. Contributions per site ranged from 13 to 105 typically-developing participants per site chosen as matched controls for the ASD population at each site. For both studies, 3 Tesla, T1-weighted MRI were acquired from 17 sites; images and acquisition details are available at http://fcon_1000.projects.nitrc.org/indi/abide. All participating sites received local Institutional Review Board approval for acquisition of the contributed data.

For further details, see Supplemental Information.

### Image processing

Quality control assessment for the PING data is detailed in Jernigan *et al*.^56^. In brief, images were inspected for excessive distortion, operator compliance, or scanner malfunction. Specifically, T1-weighted images were examined slice-by-slice for evidence of motion artefacts or ghosting and rated as acceptable, or recommended for re-scanning. After additional, on-site, visual quality control assessment, we removed a further 5 participants, resulting in a final sample of n = 768 (mean age = 12.3 y; range: 3.2–21.0 y; 404 male). Site-specific demographic data are shown in Table S1.

For all subjects, vertex-wise maps of cortical thickness and cortical area (estimated on the white matter surface) were constructed from T1 MRI with FreeSurfer 5.3 (http://surfer.nmr.mgh.harvard.edu). Briefly, this process includes removal of non-brain tissue, transformation to Talairach space, intensity normalisation, tissue segmentation and tessellation of the grey matter/white matter boundary followed by automated topology correction. Cortical geometry was matched across individual surfaces using spherical registration^59–62^. Any images that failed initial surface reconstruction, or returned surfaces with topological errors, were manually fixed using white matter mask editing and re-submitted to Freesurfer until all datasets passed inspection.

In addition, whole-brain tissue volume maps were estimated using deformation-based morphometry^63,64^. Each participant’s T1 image was intensity normalised, corrected for bias field inhomogeneities and aligned to MNI 152 space using diffeomorphic nonlinear registration (ANTs)^65,66^. Transformed images were visually inspected to ensure alignment to the template images and voxel-wise maps of volume change induced by the nonlinear deformation were characterised by the determinant of the Jacobian operator, referred to here as the Jacobian map. Each map was log-transformed so that values greater than 0 represent local areal expansion in the subject relative to the target and values less than 0 represent areal contraction.

Prior to analysis, both tissue volume maps and cortical thickness and area maps were smoothed with a Guassian kernel of 10 FWHM.

### Manifold learning

The aim of Neighbourhood Preserving Embedding is to calculate a linear transformation, *P*, to map a high-dimensional $n\times D$ dataset $X=\{{X}_{1},{X}_{2},\cdots ,\,{X}_{n}\}$ into a low-dimensional *n* × *d* subspace $Y=\{{Y}_{1},\,{Y}_{2},\,\cdots ,{Y}_{n}\}$ where $d\ll D$ and $Y={P}^{T}X$ while preserving the local neighbourhood structure of the data^17^. The process is illustrated in Fig. 1. For a given data point *X*_*i*_, an adjacency matrix is first constructed, placing an edge between *X*_*i*_ and *X*_*j*_ only if *X*_*j*_ belongs to the set of $k$ nearest neighbours of *X*_*i*_. Following this, a set of weights, *W*, is calculated that approximately reconstruct *X*_*i*_ from its neighbours and a linear projection, *P*, sought to optimally preserve this structure in the low-dimensional space, *Y* ^17^. NPE is closely related to Locally Linear Embedding^19^, but one of the major benefits that NPE confers is that the solution generalises to new datapoints, allowing unseen data to be projected onto the manifold without re-calculating the embedding.

The analysis pipeline used in this study is shown in Fig. 1C. For each image metric (tissue volume, cortical thickness, cortical area), data were first mean-centred and projected to an orthogonal subspace via singular value decomposition (SVD), while retaining 95% of variance, to reduce computational complexity and avoid overfitting. NPE was then performed using *k* = 10 neighbours, projecting data to $d=3$ dimensions. In order to maximally preserve age- and sex-related variation in the embedded data, we incorporated participant attributes into the construction of the adjacency matrices. Nearest neighbours were selected based on the product of two adjacency matrices, *A* and $a$, where the ${(i,j)}^{th}$ element of each matrix represents the (normalised) similarity between images, $A$, and age, $a$, of subjects $i$ and $j$, respectively and ${A}_{i,j}=0$, if ${S}_{i}\ne {S}_{j}$, where *S* indicates the sex of the participant. In order to reduce potential bias in image similarities due to site effects, we also introduced an additional constraint: ${A}_{i,j}=0$, if ${s}_{i}={s}_{j}$, where $s$ indicates the site/scanner of image acquisition, although this had little effect on the final embedding.

This resulted in three sets of coordinates, ${Y}_{v}$, ${Y}_{t}$, and ${Y}_{a}$, representing the low-dimensional embedding of tissue volume, $v$, cortical thickness, $t$, and area, $a$, data. We concatenate the embedding coordinates to produce a final low-dimensional representation of the combined, multi-metric image data for statistical analysis.

### Statistical analysis

Internal validity of the model was assessed using 10-fold cross-validation. We used 90% of the PING participants as a training set, calculating the manifold embedding coordinates for each imaging modality and combining them into a single representation. To predict age, we used a Gaussian Process Regression (GPR) model with the concatenated manifold coordinates as features and age as a dependent variable^67^. To predict sex, the combined coordinate set was sent to a linear discriminant classifier. Image data from the remaining 10%, the test set, were then projected onto the joint manifold and the fitted models used to predict age and sex. Mean absolute error in age estimation (MAE) and correlation between true age and predicted age are reported, alongside classification accuracies for sex. This process was repeated for each fold, reconstructing the manifold each time, such that all PING participants were part of the test set exactly once.

External validity was assessed by projecting the combined ABIDE and ABIDE-II datasets^57,58^ onto a manifold constructed from the full PING dataset and predicting age and sex using models trained on the PING data (see Supplemental Information).

To determine if errors in image-derived age estimation correlated with cognitive performance, we performed a further set of analyses using available cognitive data. Of the PING dataset, n = 617 had complete records for NTCB score, family socioeconomic status (household income and parental education), and genetic ancestry^56,68^. NTCB scores were corrected for age, sex, socioeconomic status and GAF^47^ and linear regression used to determine associations between age estimation error (based on cross-validated age predictions) and corrected cognitive scores.

All statistical analysis was performed in Matlab R2105b (Natick, MA).

### Data and code availability

PING data are available from http://pingstudy.ucsd.edu subject to a data usage agreement.

ABIDE data are available from http://fcon_1000.projects.nitrc.org/indi/abide.

The original Matlab code to perform NPE and linear graph embedding is available from http://www.cad.zju.edu.cn/home/dengcai/Data/code/.

In addition, we have made available example code to perform supervised NPE at: http://developmentalimagingmcri.github.io.

## Electronic supplementary material

Supplemental Materials

## References

[1] Dekaban, A. S. & Sadowsky, D. Changes in brain weights during the span of human life: relation of brain weights to body heights and body weights. *Ann. Neurol.* **4**, 345–356 (1978). 10.1002/ana.410040410 727739

[2] Yakovlev, P. I. & Lecours, A. R. The myelogenetic cycles of regional maturation of the brain. In *Regional Development of the Brain in Early Life* 3–69 (Blackwell, 1967).

[3] Huttenlocher PR (1979). Synaptic density in human frontal cortex - developmental changes and effects of aging. Brain Res..

[4] Mills KL (2016). Structural brain development between childhood and adulthood: Convergence across four longitudinal samples. NeuroImage.

[5] Tamnes CK (2013). Brain development and aging: Overlapping and unique patterns of change. NeuroImage.

[6] Aubert-Broche B (2013). A new method for structural volume analysis of longitudinal brain MRI data and its application in studying the growth trajectories of anatomical brain structures in childhood. NeuroImage.

[7] Lebel C, Beaulieu C (2011). Longitudinal Development of Human Brain Wiring Continues from Childhood into Adulthood. J. Neurosci..

[8] Tamnes, C. K. *et al*. Development of the Cerebral Cortex across Adolescence: A Multisample Study of Inter-Related Longitudinal Changes in Cortical Volume, Surface Area, and Thickness. *J. Neurosci.* **37**, 3402–3412 (2017). 10.1523/JNEUROSCI.3302-16.2017 PMC5373125 28242797

[9] Sowell ER (2007). Sex differences in cortical thickness mapped in 176 healthy individuals between 7 and 87 years of age. Cereb. Cortex N. Y. N 1991.

[10] Koolschijn PCMP, Crone EA (2013). Sex differences and structural brain maturation from childhood to early adulthood. Dev. Cogn. Neurosci..

[11] Mutlu AK (2013). Sex differences in thickness, and folding developments throughout the cortex. NeuroImage.

[12] Giedd JN (1999). Brain development during childhood and adolescence: a longitudinal MRI study. Nat. Neurosci..

[13] Lenroot RK (2007). Sexual dimorphism of brain developmental trajectories during childhood and adolescence. NeuroImage.

[14] Fjell AM (2010). When does brain aging accelerate? Dangers of quadratic fits in cross-sectional studies. NeuroImage.

[15] Mills KL, Tamnes CK (2014). Methods and considerations for longitudinal structural brain imaging analysis across development. Dev. Cogn. Neurosci..

[16] Paus T, Wong AP-Y, Syme C, Pausova Z (2017). Sex differences in the adolescent brain and body: Findings from the Saguenay youth study. J. Neurosci. Res..

[17] He, X., Cai, D., Yan, S. & Zhang, H.-J. Neighborhood preserving embedding. In *Tenth IEEE International Conference on Computer Vision (ICCV’05)**Volume 1* **2**, 1208–1213 (2005).

[18] He X, Yan S, Hu Y, Niyogi P, Zhang H-J (2005). Face recognition using Laplacianfaces. IEEE Trans. Pattern Anal. Mach. Intell..

[19] Roweis ST, Saul LK (2000). Nonlinear dimensionality reduction by locally linear embedding. Science.

[20] Tenenbaum JB, de Silva V, Langford JC (2000). A global geometric framework for nonlinear dimensionality reduction. Science.

[21] Lerman G, Shakhnovich BE (2007). Defining functional distance using manifold embeddings of gene ontology annotations. Proc. Natl. Acad. Sci..

[22] Yao F, Coquery J, Lê Cao K-A (2012). Independent Principal Component Analysis for biologically meaningful dimension reduction of large biological data sets. BMC Bioinformatics.

[23] You Z-H, Lei Y-K, Gui J, Huang D-S, Zhou X (2010). Using manifold embedding for assessing and predicting protein interactions from high-throughput experimental data. Bioinformatics.

[24] Zeng, X. & Luo, S. A Supervised Subspace Learning Algorithm: Supervised Neighborhood Preserving Embedding. *Advanced Data Mining and Applications* 81–88 (2007).

[25] Panta, S. R. *et al*. A Tool for Interactive Data Visualization: Application to Over 10,000 Brain Imaging and Phantom MRI Data Sets. *Front. Neuroinformatics* **10**, (2016). 10.3389/fninf.2016.00009 PMC4791544 27014049

[26] Wolz, R., Aljabar, P., Hajnal, J. V. & Rueckert, D. Manifold Learning for Biomarker Discovery in MR Imaging. In *Machine Learning in Medical Imaging* 116–123 Springer, Berlin, Heidelberg (2010).

[27] McKeown MJ (1998). Analysis of fMRI data by blind separation into independent spatial components. Hum. Brain Mapp..

[28] Liu, X., Tosun, D., Weiner, M. W. & Schuff, N. Locally Linear Embedding (LLE) for MRI based Alzheimer’s Disease Classification. *NeuroImage* **83**, (2013). 10.1016/j.neuroimage.2013.06.033 PMC3815961 23792982

[29] Aljabar P (2011). A combined manifold learning analysis of shape and appearance to characterize neonatal brain development. IEEE Trans. Med. Imaging.

[30] Kadoury, S., Erus, G., Zacharaki, E. I., Paragios, N. & Davatzikos, C. Manifold-constrained embeddings for the detection of white matter lesions in brain MRI. In *2012 9th IEEE International Symposium on Biomedical Imaging (ISBI)* 562–565 (2012). 10.1109/ISBI.2012.6235610 PMC3892901 24443675

[31] Brown TT (2012). Neuroanatomical assessment of biological maturity. Curr. Biol. CB.

[32] Dosenbach NUF (2010). Prediction of Individual Brain Maturity Using fMRI. Science.

[33] Erus G (2015). Imaging Patterns of Brain Development and their Relationship to Cognition. Cereb. Cortex.

[34] Franke K, Ziegler G, Klöppel S, Gaser C (2010). Estimating the age of healthy subjects from T1-weighted MRI scans using kernel methods: Exploring the influence of various parameters. NeuroImage.

[35] Khundrakpam BS, Tohka J, Evans AC (2015). Prediction of brain maturity based on cortical thickness at different spatial resolutions. NeuroImage.

[36] Chekroud AM, Ward EJ, Rosenberg MD, Holmes AJ (2016). Patterns in the human brain mosaic discriminate males from females. Proc. Natl. Acad. Sci. USA.

[37] Rosenblatt JD (2016). Multivariate revisit to ‘sex beyond the genitalia’. Proc. Natl. Acad. Sci. USA.

[38] Franke K, Luders E, May A, Wilke M, Gaser C (2012). Brain maturation: predicting individual BrainAGE in children and adolescents using structural MRI. NeuroImage.

[39] Hofmann T, Schölkopf B, Smola AJ (2008). Kernel Methods in Machine Learning. Ann. Stat..

[40] Sowell ER (2004). Longitudinal Mapping of Cortical Thickness and Brain Growth in Normal Children. J. Neurosci..

[41] Xie Y, Chen YA, De Bellis MD (2012). The relationship of age, gender, and IQ with the brainstem and thalamus in healthy children and adolescents: a magnetic resonance imaging volumetric study. J. Child Neurol..

[42] Amlien IK (2016). Organizing Principles of Human Cortical Development—Thickness and Area from 4 to 30 Years: Insights from Comparative Primate Neuroanatomy. Cereb. Cortex.

[43] Fjell AM (2015). High-Expanding Cortical Regions in Human Development and Evolution Are Related to Higher Intellectual Abilities. Cereb. Cortex.

[44] Giedd JN, Raznahan A, Mills KL, Lenroot RK (2012). Review: magnetic resonance imaging of male/female differences in human adolescent brain anatomy. Biol. Sex Differ..

[45] Bramen JE (2012). Sex Matters during Adolescence: Testosterone-Related Cortical Thickness Maturation Differs between Boys and Girls. PLoS ONE.

[46] Cole JH, Franke K (2017). Predicting Age Using Neuroimaging: Innovative Brain Ageing Biomarkers. Trends Neurosci..

[47] Akshoomoff N (2014). TheNIH Toolbox Cognition Battery: Results from a Large Normative Developmental Sample (PING). Neuropsychology.

[48] Han, C. E., Peraza, L. R., Taylor, J. P. & Kaiser, M. Predicting age across human lifespan based on structural connectivity from diffusion tensor imaging. In *2014 IEEE Biomedical Circuits and Systems Conference (BioCAS) Proceedings* 137–140 (2014).

[49] Liem F (2017). Predicting brain-age from multimodal imaging data captures cognitive impairment. NeuroImage.

[50] Beckmann CF, Smith SM (2004). Probabilistic independent component analysis for functional magnetic resonance imaging. IEEE Trans. Med. Imaging.

[51] O’Muircheartaigh, J. & Jbabdi, S. Concurrent white matter bundles and grey matter networks using independent component analysis. *NeuroImage*10.1016/j.neuroimage.2017.05.012 (2017). 10.1016/j.neuroimage.2017.05.012 PMC6318261 28514668

[52] Martinez-Murcia FJ (2017). On the brain structure heterogeneity of autism: Parsing out acquisition site effects with significance-weighted principal component analysis. Hum. Brain Mapp..

[53] Maaten Lvd, Hinton G (2008). Visualizing Data using t-SNE. J. Mach. Learn. Res..

[54] Plis, S. M. *et al*. Deep learning for neuroimaging: a validation study. *Front. Neurosci*. **8** (2014). 10.3389/fnins.2014.00229 PMC4138493 25191215

[55] Cole JH (2017). Predicting brain age with deep learning from raw imaging data results in a reliable and heritable biomarker. NeuroImage.

[56] Jernigan TL (2016). The Pediatric Imaging, Neurocognition, and Genetics (PING) Data Repository. NeuroImage.

[57] Di Martino A (2014). The autism brain imaging data exchange: towards a large-scale evaluation of the intrinsic brain architecture in autism. Mol. Psychiatry.

[58] Di Martino A (2017). Enhancing studies of the connectome in autism using the autism brain imaging data exchange II. Sci. Data.

[59] Dale AM, Fischl B, Sereno MI (1999). Cortical Surface-Based Analysis: I. Segmentation and Surface Reconstruction. NeuroImage.

[60] Fischl B (2002). Whole brain segmentation: automated labeling of neuroanatomical structures in the human brain. Neuron.

[61] Fischl B, Sereno MI, Dale AM (1999). Cortical Surface-Based Analysis: II: Inflation, Flattening, and a Surface-Based Coordinate System. NeuroImage.

[62] Fischl B, Dale AM (2000). Measuring the thickness of the human cerebral cortex from magnetic resonance images. Proc. Natl. Acad. Sci. USA.

[63] Ashburner J (1998). Identifying global anatomical differences: deformation-based morphometry. Hum. Brain Mapp..

[64] Rueckert, D., Frangi, A. F. & Schnabel, J. A. Automatic construction of 3-D statistical deformation models of the brain using nonrigid registration. *IEEE Trans. Med. Imaging* **22**, 1014–1025 (2003). 10.1109/TMI.2003.815865 12906255

[65] Avants BB, Epstein CL, Grossman M, Gee JC (2008). Symmetric diffeomorphic image registration with cross-correlation: evaluating automated labeling of elderly and neurodegenerative brain. Med. Image Anal..

[66] Tustison NJ (2010). N4ITK: Improved N3 Bias Correction. IEEE Trans. Med. Imaging.

[67] Cole JH, Leech R, Sharp DJ (2015). Alzheimer’s Disease Neuroimaging Initiative. Prediction of brain age suggests accelerated atrophy after traumatic brain injury. Ann. Neurol..

[68] Libiger O, Schork NJ (2012). A Method for Inferring an Individual’s Genetic Ancestry and Degree of Admixture Associated with Six Major Continental Populations. Front. Genet..

