# Supplementary material for: Modelling neuroanatomical variation during childhood and adolescence with neighbourhood-preserving embedding
Source: Sci Rep. 2017 Dec 19;7:17796. doi: 10.1038/s41598-017-18253-6 (PMC5736651; doi:10.1038/s41598-017-18253-6)
Supplement: Supplementary file 1 — Supplemental Materials [file 41598_2017_18253_MOESM1_ESM.pdf]

Modelling neuroanatomical variation during childhood and adolescence with neighbourhood-preserving embedding

Gareth Ball[1], Chris Adamson[1], Richard Beare[1] and Marc L. Seal[1,2]

1. Developmental Imaging, Murdoch Children's Research Institute.
2. Department of Paediatrics, University of Melbourne

Corresponding author:

Dr Gareth Ball
Developmental Imaging,
Murdoch Children's Research Institute,
The Royal Children's Hospital,
Flemington Road
Parkville 3052
Melbourne, Australia

gareth.ball@mcri.edu.au

Supplemental Information

ABIDE and ABIDE-II

External validation data was collated from the freely available ABIDE and ABIDE-II cohorts.

The ABIDE cohort included imaging data from 1112 participants. After initial visual quality assessment, 18 were removed due to motion artefacts and 10 due to poor image contrast. A further 28 were removed due to prior pre-processing (n=1), or high similarity to other images within ABIDE, or ABIDE-II (i.e.: repeat scans, with similar participants age and same sex). Remaining T1 images from typically-developing participants aged 21y and under from 17 sites were included in the final sample of n=424 (346 male; mean age=13.69y).

In total, n=1044 datasets were available to download from ABIDE-II. Of these, 28 were excluded due to visible image artefacts, and a further 13 failed cortical reconstruction. Two images appeared to have corresponding repeat scans and were removed. As above, we removed those aged over 21, and those with an ASD diagnosis, resulting in a final sample of n=439 (297 male; mean age=11.50y) participants from 15 sites. Site-specific demographic data for ABIDE and ABIDE-II are shown in Table S2.

In total, 13 ABIDE and 16 ABIDE-II images required manual editing to complete surface reconstruction in Freesurfer.

Supplemental Tables and Figures

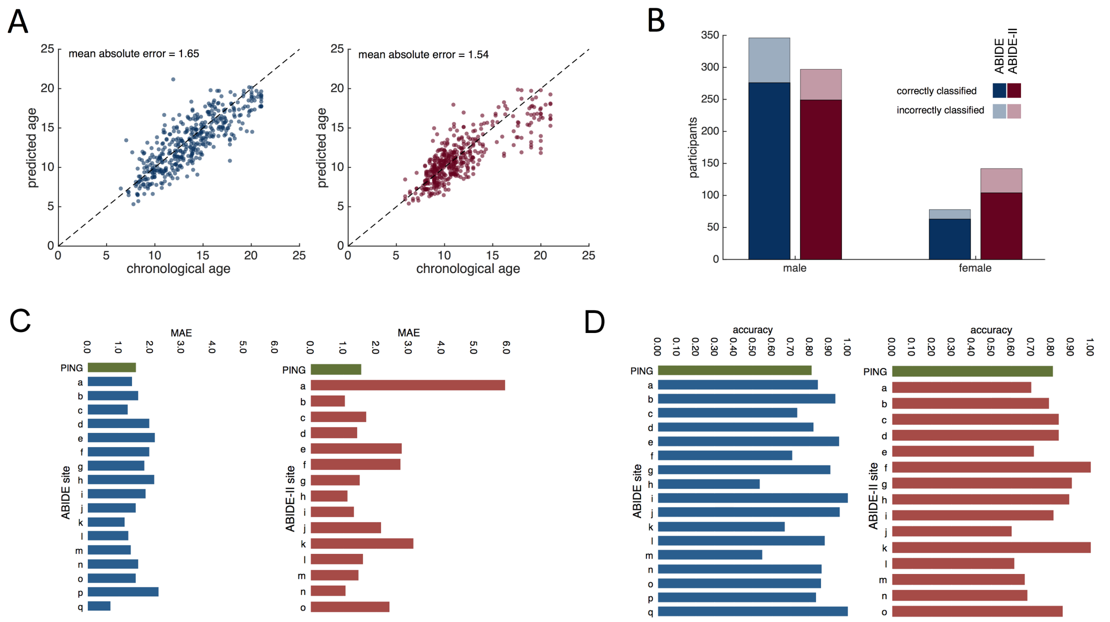

Figure S1: Model accuracy in an independent dataset. A. Scatterplots show the correlation between true and predicted age in the ABIDE cohorts, using a model trained with the PING dataset. B. Sex classification accuracies for ABIDE and ABIDE-II. Note that accuracy was similar in male and female populations despite the unbalanced sex distributions. C. MAE for each site in ABIDE and ABIDE-II, D. Corresponding sex classification accuracies.

Table S1: PING demographics by site

Site	N	Age (range)	Male (%)
a	112	15.14 (4.08-21.0)	57 (50.8)
b	105	14.69 (3.75-21.0)	59 (56.2)
c	118	12.29 (3.17-21.0	58 (49.2)
d	124	12.03 (3.17-20.92)	68 (54.8)
e	64	13.42 (4.17-20.92)	34 (53.1)
f	129	8.79 (3.25-20.25)	65 (50.4)
g	116	10.87 (3.42-21.00)	63 (54.3)
Total	768	12.28 (3.17-21.0)	404 (52.6)

Table S2: ABIDE and ABIDE-II demographics by site

ABIDE Site	N	Age (range)	Male (%)
a	19	15.30 (9.44-20.65)	16 (84.2)
b	15	16.53 (10.0-21.0)	13 (86.7)
c	15	10.06 (8.20-11.99)	15 (100)
d	22	14.22 (8.67-16.88)	16 (72.8)
e	21	16.28 (12.04-20.33)	21 (100)
f	72	14.48 (8.2-19.2)	54 (75.0)
g	22	15.31 (8.77-19.76)	22 (100)
h	28	12.68 (7.66-17.83)	20 (71.4)
i	4	20.75 (20.0-21.0)	3 (75.0)
j	23	15.08 (12.20-21.0)	18 (78.2)
k	24	10.01 (8.07-12.77)	20 (83.3)
l	82	13.08 (6.47-20.56)	63 (76.8)
m	20	9.95 (7.75-12.43)	16 (80.0)
n	43	12.96 (9.21-17.79)	37 (86.0)
o	7	12.57 (7.00-21.00)	7 (100)
p	6	19.52 (17.0-20.90)	4 (66.7)
q	1	20 (-)	1 (-)
Total	424	13.69 (6.47-21.0)	346 (81.6)

ABIDE-II Site	n	age (range)	male (%)
a	10	19.6 (18.0-21.0)	7 (80.0)
b	52	10.45 (8.06-13.80)	26 (50.0)
c	25	13.25 (8.10-17.70)	23 (92.0)
d	56	10.38 (8.0-14.0)	27 (48.2)
e	7	19.43 (18.0-21.0)	7 (100)
f	6	17.89 (13.83-20.17)	6 (100)
g	21	15.61 (10.25-20.0)	21 (100)
h	28	9.06 (5.89-12.9)	27 (96.4)
i	154	10.35 (8.02-12.90)	99 (64.2)
j	5	14.79 (11.50-18.58)	5 (100)
k	8	20.25 (19.0-21.0)	6 (75.0)
l	12	14.30 (8.17-20.51)	3 (25.0)
m	15	9.81 (7.76-14.09)	10 (66.7)
n	25	8.15 (6.33-10.12)	20 (80.0)
o	14	14.80 (12.25-17.17)	10 (71.4)
Total	438	11.5 (5.89-21.00)	297 (67.7)
